# Brunsting-Perry Pemphigoid Confined to the Scalp: A Clinicopathologic Mimicker of Discoid Lupus and Central Centrifugal Cicatricial Alopecia (CCCA)

**DOI:** 10.7759/cureus.114753

**Published:** 2026-08-18

**Authors:** Danielle S Jaffe, Nicole Alhov, Blake C Van Noord, Michelle Sobotka, Benjamin Kahn, Dionne Louis, Marcus B Goodman

**Affiliations:** 1 Medical School, Tulane University School of Medicine, New Orleans, USA; 2 Medical School, Philadelphia College of Osteopathic Medicine, Philadelphia, USA; 3 Dermatology, Goodman Dermatology, Roswell, USA; 4 Dermatology, Leading Edge Dermatology, Plantation, USA

**Keywords:** autoimmune blistering disease, brunsting-perry pemphigoid, bullous pemphigoid, scarring alopecia, subepidermal blistering disorders

## Abstract

Brunsting-Perry pemphigoid (BPP) is a rare variant of cicatricial pemphigoid that is difficult to diagnose due to its overlapping clinical and histopathologic features with other causes of scarring alopecia. Due to the rarity of this condition, to date, less than 100 cases have been documented in the literature. We present a 76-year-old female with a four-month history of painful, scaly lesions on the scalp, whose differential diagnosis originally included discoid lupus erythematosus and central centrifugal cicatricial alopecia due to their ability to mimic BPP. The diagnostic overlap in this case highlights how autoimmune blistering diseases like BPP can mimic both inflammatory dermatoses and primary cicatricial alopecias, reinforcing the need for careful clinicopathologic correlation to avoid misdiagnosis and to better guide treatment.

## Introduction

Brunsting-Perry pemphigoid (BPP) is a rare variant of cicatricial pemphigoid, a localized autoimmune blistering disorder that targets hemidesmosomal proteins. Originally identified by Brunsting and Perry in 1957, BPP is a rare subepidermal blistering disease that only affects the scalp, face, and neck, with minimal or no mucosal involvement [1,2]. Clinically, BPP is characterized by tense, pruritic bullae in these regions that heal with atrophic scarring [3]. Its clinical and histopathologic features can overlap with other causes of scarring alopecia, including discoid lupus erythematosus (DLE), central centrifugal cicatricial alopecia (CCCA), and epidermolysis bullosa acquisita (EBA), posing a considerable diagnostic challenge. To date, less than 100 cases have been documented in the literature, underscoring its rarity and the potential for clinical misdiagnosis [2].

We present a case of BPP initially misdiagnosed as DLE due to its clinical presentation. However, subsequent histopathologic findings later prompted consideration of CCCA, a subepidermal blistering disease, versus cicatricial pemphigoid. This case of BPP demonstrates the diagnostic complexity of BPP, which can mimic both autoimmune and primary scarring alopecia. Misdiagnosis of BPP leads to delayed treatment and further progression of the disease, which is harmful to the patient. Moreover, this case emphasizes the importance of integrating clinical and histologic data when evaluating scarring scalp lesions.

## Case presentation

**Figure 1:**
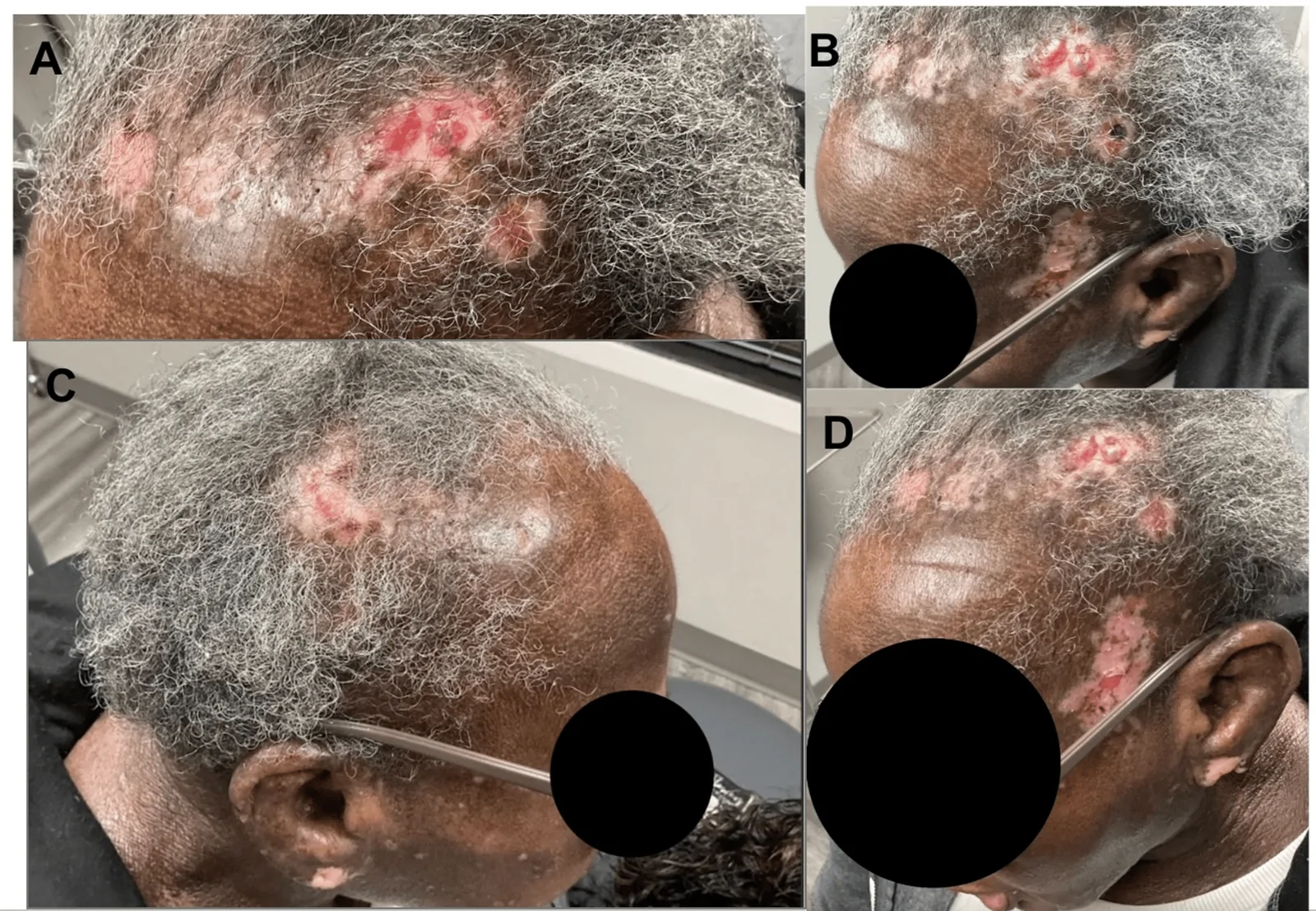
Brunsting-Perry Pemphigoid Well-demarcated, hypopigmented, atrophic plaques with overlying scale and erosions on the left frontal and temporal scalp in a 76-year-old woman.

A 76-year-old female with a history of type 2 diabetes mellitus, arthritis, asthma, chronic obstructive pulmonary disease (COPD), hypertension, and hypercholesterolemia presented with a four-month history of painful, scaly lesions on the scalp as seen in Figure 1. She reported no history of autoimmune disease. She noted that the lesions were tender, non-healing, and occasionally pustular. The patient reported that the onset of these lesions coincided with the use of a curl cream/conditioner, which she believed may have triggered an allergic reaction. She had been self-treating the lesions with Vaseline and Sulfur8 medicated products, while resorting to wearing wigs to conceal her condition. 

On physical exam, well-demarcated atrophic plaques with overlying scale and hypopigmented alopecic patches were noted on the left frontal scalp and forehead (Figure 1). An initial clinical differential diagnosis included alopecia versus alopecia secondary to DLE. Punch biopsies of the left lateral forehead and left central frontal scalp were performed and stained with hematoxylin and eosin. Histopathology revealed inflammatory scarring alopecia with grade 3/3 inflammation with eosinophils and plasma cells. Subepidermal blistering both at the surface and around deep hair follicles was also noted (Figure 1). Additionally, the report showed evidence of perifollicular fibrosis and polytrichia. The histopathological differential diagnosis given included advanced CCCA as well as cicatricial pemphigoid, specifically the localized BPP variant. Direct immunofluorescence (DIF) was recommended to confirm the diagnosis, but not considered necessary due to the other clinicopathologic findings present and no further immunologic studies were performed. We favor the BPP variant of bullous pemphigoid not only due to the clinical presentation, but also due to the pathology, which showed fibrosis and an infiltrate that includes eosinophils.

The patient was started on a 12-day prednisone taper starting at 40mg for initial preliminary treatment. Topical treatments included clobetasol 0.05% solution for the scalp, triamcinolone 0.025% cream for the face, and tacrolimus 0.1% ointment to use on days off from the steroid. The patient did not return for a follow-up as she had no new concerns and did not have primary means of transportation to and from our clinic. Due to the loss of follow-up, no other immunosuppressive drugs were prescribed as they would require a more extensive workup. 

## Discussion

Due to the rarity of BPP and its overlapping clinical and histologic features with more common scalp disorders, BPP is often misdiagnosed, potentially delaying appropriate treatment. In this case, DIF was not pursued in an effort to reduce the burden of additional testing, particularly given that DIF produces false negatives in detecting peripheral autoantibodies [2,4]. DIF can detect many different basement membrane antigens, producing a false negative test, so it could lead to an incorrect diagnosis of BPP, and the diagnosis of BPP is not dependent on this test [2,5]. The diagnosis of BPP relies on the clinical and histopathological findings for each case [2]. Given the clinicopathologic findings present in this case, including subepidermal blistering with eosinophils and local scarring alopecia distribution along the head and neck excluding other entities, a DIF was not necessary as it is not required to diagnose BPP. The careful integration of the clinical and histopathologic findings allowed for clinical diagnosis of BPP while minimizing both patient cost and burden. 

The patient’s initial clinical presentation of erythematous, scaly lesions with alopecia was suggestive of DLE. Both DLE and BPP can present with scarring alopecic plaques on the scalp and can share symptoms such as pruritus and tenderness [3,6]. DLE most often appears as scaly plaques, exhibiting follicular plugging and photosensitivity that is exacerbated by sun exposure [7]. On the other hand, BPP more often presents with tense bullae or erosions, but can have overlying scale, which can contribute to diagnostic confusion [8]. Given the diagnostic overlap, similar cases of cicatricial pemphigoid have been misdiagnosed as DLE [9]. Although these conditions overlap with clinical presentation in many ways, their histopathology differs since DLE is associated with interface dermatitis while BPP is associated with a subepidermal blister and the presence of eosinophils (Table 1). 

**Table 1:** Clinical differentiations of dermal conditions that lead to scarring alopecia

Condition	Clinical features	Key Histopathology	Target antigen
Discoid lupus erythematosus (DLE)	Well-demarcated plaques with scarring alopecia [7]	Interface dermatitis [7]	Keratinocyte-associated proteins [7]
Centrifugal cicatricial alopecia (CCCA)	Hair loss with erythema and pustules [10]	Perifollicular fibrosis [10]	Hair follicle [10]
Brungsting-Perry pemphigoid (BPP)	Tense, pruritic bullae with atrophic scarring on the head, neck, and scalp [12]	Eosinophilic with subepidermal blister [12]	Hemidesmosomes (BP180, BP230) [12]
Epidermolysis bullosa acquisita (EBA)	Scarring milia found on extensor surfaces [13]	Subepidermal blister [13]	Type VII collagen [13]

Immunopathologic findings did not show any features consistent with DLE. Rather, the biopsy raised suspicion for CCCA due to the presence of perifollicular fibrosis and polytrichia - a pattern commonly seen in primary scarring alopecias [10]. However, the presence of eosinophils, which is characteristic of BPP, reflected an ongoing immunologic process that targets the dermoepidermal junction [11]. The subepidermal blistering, along with the presence of eosinophils, is characteristic of BPP and distinguishes it from primary alopecias such as CCCA [4,8]. Additionally, in clinic the lesion morphology was not consistent with CCCA. CCCA typically presents as symmetric, diffuse thinning at the vertex with centrifugal progression, rather than the scaly, well-demarcated plaques that were observed [10]. While these two conditions may share a common pattern associated with scarring alopecias, CCCA targets the hair follicle while BPP targets hemidesmosomes (Table 1). The patient's history of type 2 diabetes also supports the diagnosis of BPP. While EBA is a scarring alopecia that presents with similarities to BPP, such as the presence of a subepidermal blister, they differ in clinical presentation and target antigens (Table 1). EBA tends to present on extensor surfaces, while BPP is present on the head, neck, and scalp. Additionally, EBA targets type VII collagen while BPP targets hemidesmosomes (Table 1). In this case the lesions were localized to the head, neck, and scalp, therefore the clinical presentation was not suggestive of EBA. 

## Conclusions

This case underscores the importance of integrating lesion morphology, symptom profile, and histologic appearance when evaluating scarring scalp disorders. Although the clinical presentation was initially suggestive of DLE, histopathology introduced CCCA as a possible diagnosis despite clinical features not supporting it. The absence of DIF confirmation may pose a limitation in a confident diagnosis of BPP; however, due to the variability of DIF and presence of multiple clinicopathologic findings, a case of BPP is highly suggestive. The diagnostic overlap in this case highlights how autoimmune blistering diseases like BPP can mimic both inflammatory dermatoses and primary cicatricial alopecias, reinforcing the need for careful clinicopathologic correlation to avoid misdiagnosis and to better guide treatment. 
